# Application of the Chinese Version of the BIS/BAS Scales in Participants With a Substance Use Disorder: An Analysis of Psychometric Properties and Comparison With Community Residents

**DOI:** 10.3389/fpsyg.2020.00912

**Published:** 2020-05-08

**Authors:** Qingqing Che, Peiwen Yang, Huiyuan Gao, Meizhu Liu, Jun Zhang, Taisheng Cai

**Affiliations:** ^1^Medical Psychological Center, Second Xiangya Hospital, Central South University, Changsha, China; ^2^Medical Psychological Institute, Central South University, Changsha, China; ^3^National Clinical Research Center for Mental Disorders, Changsha, China; ^4^Hunan Judicial Police Vocational College, Changsha, China

**Keywords:** Behavioral Inhibition, Behavioral Activation, people with a substance use disorder, psychometric properties, impulsivity, self-control ability

## Abstract

Carver and White developed the Behavioral Inhibition/Behavioral Activation Scales (the BIS/BAS Scales) based on Reinforcement Sensitivity Theory proposed by Gray. Subsequent studies proposed that substance abuse was closely related to Behavioral Inhibition System (BIS) and Behavioral Activation System (BAS). However, researches on the psychometric properties of the BIS/BAS scales in clinical samples are scarce. The present study was conducted to analyze the applicability of the BIS/BAS scales in a sample suffering from a substance use disorder (SUD) and undergoing treatment in compulsory detoxification institutions (*n* = 1117). Meanwhile, 822 community residents were selected for comparison. Confirmatory factor analysis was carried out to examine the construct validity and the results showed that the five-factor model was the best fit for people with a substance use disorder’ data. Besides, Cronbach’s alpha coefficient for the total scale was 0.808, indicating the satisfactory internal consistency reliability. Analysis of the correlation coefficient of the questionnaire with the corresponding personality traits showed that BAS was more associated with the impulsive trait. Surprisingly, participants with a substance use disorder showed more insensitivity for the reward dimension compared with that of community residents and the result of comparison between two samples supported joint subsystems hypothesis. Generally, the BIS/BAS scales showed good reliability and validity. These findings provide more direct evidence on the personality traits of people with a substance use disorder and should form the basis for further research.

## Introduction

Drug abuse is a public health and social issue worldwide. Studies have shown that drugs cause serious harm to individuals. They not only lead to deficits in response inhibition (Bell et al., 2014; Smith et al., 2014), but also damage the execution function and make people more impulsive (Dolan et al., 2008). Neuroimaging results showed that the prefrontal cortex and other structures involved in higher-order executive function (such as self-control) of individuals with drug addiction were damaged by compulsive drug taking (Goldstein and Volkow, 2011). Drugs damage the physiological structure of drug users as well as affecting their mental health. Relevant surveys found that most substance users suffer from marked mental pressure (Andreas et al., 2015), and were prone to psychological problems (Schuckit and Hesselbrock, 1994; Gilbert, 2000), such as anxiety and depression. Therefore, anxious and impulsive personality traits were considered as risk factors for drug addiction (Belin et al., 2008; Ersche et al., 2012).

The personality characteristics of anxiety and impulsivity have been studied widely. The reinforced sensitivity theory proposed by Gray (1982, 1987) was one of the most influential biological personality theories (Yu et al., 2011) in this field. The theory postulated that anxiety and impulsivity were the two main dimensions of personality, which are in an orthogonal relationship and are regulated by the behavioral Inhibition scale (BIS) and behavioral Activation scales (BAS), respectively. BIS is responsible for dealing with the processing of target conflicts. When a stimulus that triggers the target conflict is presented, this system is activated and is accompanied by the generation of anxiety. Then, the system will suppress the originally dominant behavior in the conflict and make people evaluate the gains and losses to find the best way, in memory and context, to resolve the conflict. BAS is sensitive to all appetitive stimuli (conditional and unconditional). When presenting reward signals or withdrawing punishment signals, BAS is activated, accompanied by the generation of positive emotions to promote ongoing behavior (Gray and McNaughton, 2000). Carver and White (1994) developed the BIS/BAS scales based on Gray’s theory, which is the most widely used scales based on the RST (Carver and White, 1994).

To date, the BIS/BAS scales have been widely used in different countries. For instance, Caci et al. found that the BIS/BAS scales were a reliable measure in French college students (Caci et al., 2007). Beck et al. demonstrated that the BIS/BAS scales had a satisfactory construct validity and acceptable internal consistency in an analysis of German eating disordered outpatients (Beck et al., 2009). Beside these, the BIS/BAS scales have been shown to have excellent psychometric properties among other samples, such as normal school children (Muris et al., 2005) and community samples (Jorm et al., 1999). In terms of factor structure, most previous studies have supported the view that the BIS/BAS scales have a four-factor structure, named the behavior inhibition, the fun-seeking, the reward responsiveness, and the drive respectively (Carver and White, 1994). Meanwhile, many scholars have proposed certain new structures. Johnson et al. proposed a five-factor structure (Johnson et al., 2003), which was verified in a large sample of offenders (Caci et al., 2007). Heym et al. proposed another five-factor structure (Heym et al., 2008), which was confirmed in heroin-dependent participants (Dissabandara et al., 2012). The two five-factor structures mentioned above share the same three dimensions of the fun-seeking, the reward responsiveness and the drive, with different BIS-fear and the BIS-anxiety dimensions. Besides, previous studies have also analyzed one-factor structure (Müller and Wytykowska, 2005) and two-factor structure (Ross et al., 2002). The two-factor structure included two dimensions: the Behavioral Approach System and the Behavioral Inhibition System.

Current research indicates that BIS and BAS might be associated with substance abuse. Franken et al. found that the number of college students’ illegal substances use correlated positively with BAS and negatively with BIS personality characteristics (Franken and Muris, 2006). Franken et al. also found that the BAS scores were significantly higher in clinically referred drug addicts than in the control group (Franken et al., 2006). Stenason et al. found that behavioral inhibition correlated negatively with substance use (Stenason and Vernon, 2016). Meanwhile, many scholars found that substance addicts have higher impulsivity levels (Wong et al., 2013). Studies also showed that subjects with a history of drug-dependence were more likely to be impulsive than subjects with no drug-dependence history (Holdich et al., 1998). Thus, we hypothesized that people with a substance use disorder could have higher levels of BAS and lower levels of BIS than community residents. To prove this hypothesis, we compared those two personality traits between participants with a substance use disorder and a community sample on the dimensions of the scale. In addition, there should be people with four combinations of high and low BIS and BAS sensitivity within a given population according to previous studies (Carver and White, 1994) and joint subsystems hypothesis (Corr, 2002). Therefore, we further compared the difference in the number of people between the four groups in the two samples.

Previous studies have provided important information about BIS and BAS among people from different countries (Cooper et al., 2007), different ages (Yu et al., 2011), and even those with different diseases (Beck et al., 2009); however, relatively few studies have focused on people with a substance use disorder who were undergoing treatment in compulsory detoxification institutions in China and in those studies, the samples were not sufficiently representative (Franken et al., 2006). The present study is the first to test the suitability of the BIS/BAS scales in a large SUD sample in China. The authors also hope to further clarify the relationship among BIS, BAS, impulsivity and self-control ability. In general, the results of the present study could not only enrich our understanding of BIS and BAS, but also reveal the differences between two groups in terms of personality traits.

## Materials and Methods

### Samples

The inclusion criteria were as follows: The subjects met Diagnostic and Statistical Manual of Mental Disorders, Fifth Edition (DSM-V) criteria for substance use disorders and completed acute detoxification treatment; had at least 4 years of formal education; and demonstrated drug addiction. The exclusion criteria were as follows: The subjects suffered from a serious infectious disease, somatic disease, or serious mental illness; their educational level was too low to complete the scale independently; or they were unwilling to participate. The study was conducted using the convenient sampling method. The participants were people with a substance use disorder from six compulsory detoxification centers in Hunan Province. To compare the differences between the different samples on the BIS/BAS scales, community residents were also tested. Finally, we obtained valid data for 1117 participants with a substance use disorder and 822 community residents after eliminating invalid questionnaires (missing answers or false answers). In addition, we randomly selected 362 participants with a substance use disorder to complete the SPSRQ, the SCS, and the SUPPS-P. Retesting was performed after 6 weeks. 163 questionnaires were completed effectively.

### Procedure

Before the test, illiteracy was eliminated by inquiry and self-reporting, then the content, purpose, and requirement of this study was explained to each participant. Data was only collected after signed, informed consent was obtained. We respected the participants’ wishes and they could quit at any time. The study was approved by the Local Ethics Committee of our institution.

### Measures

#### The Behavioral Inhibition/Behavioral Activation Scales

The BIS/BAS scales were developed by Carver and White in 1994 with a total of 24 self-reported items and four filler items, without scoring. The scale was divided into the behavior inhibition scale (items 2, 8, 13, 16, 19, 22, 24) and the behavior activation scale. BAS comprises three subscales: The fun-seeking subscale (items 3, 9, 12, 21), the reward responsiveness subscale (items 5, 10, 15, 20), and the drive subscale (items 4, 7, 14, 18, 23). The scales were scored on a four point Likert scale (1 indicating strongly agree and 4 indicating strongly disagree). Item 2 and item 22 were reverse scoring. The lower the score, the greater the sensitivity levels of BIS or BAS. Studies have shown that the BIS/BAS scales have good reliability and validity. The internal consistency coefficient of the BIS scale and the three subscales of BAS scale ranged from 0.66 to 0.76. The corresponding test-retest reliabilities were 0.66, 0.66, 0.59, and 0.69 (Carver and White, 1994).

First, two bilingual graduate students (one each from the Psychology and English Departments) translated the BIS/BAS scales from English into Mandarin. Second, with no prior knowledge of the English version of the BIS/BAS scales, a psychologist and a linguist translated the preliminary Chinese version back into English. Third, the research team compared and discussed each item of the Chinese manuscript and the English manuscript to form the first draft of a Chinese translation. Finally, the back-translated version was reviewed by a psychology doctor with an English background to form the final draft. We obtained permission for this process from the original author.

### The Punishment and Reward Sensitivity Scales (the SPSRQ Scales)

The questionnaire was developed by Torrubia et al. (2001) and comprises a 48-item questionnaire designed to assess punishment sensitivity and reward sensitivity. The odd items formed the punishment sensitivity subscale and the even items formed the reward sensitivity subscale. The scale was scored on a two-point scale (1 indicating yes and 2 indicating no). The internal consistency coefficients of the two subscales were 0.83 and 0.78 and the retest reliabilities of the three-month interval were 0.89 and 0.87 respectively. The results of the validity study were good. In the current study, the Cronbach α of the total scale was 0.861.

#### The Short Version of the UPPS-P Impulsive Behavior Scale (the SUPPS-P Scale)

The short version of the UPPS-P impulsive behavior scale has five dimensions, including feeling seeking, lack of persistence, lack of predictability, negative urgency, and positive urgency (Cyders et al., 2014). The Cronbach α ranged from.74 to.85, suggesting acceptable to good internal consistency for the various subscales. The SUPPS-P scale has been translated and introduced into French (Billieux et al., 2012), Italian (D’Orta et al., 2015), and other versions. The results of these studies confirmed that it had good reliability and validity, and a good predictive performance for alcohol abuse and drug abuse. The Cronbach α in this study was 0.769.

#### The Self-Control Scale (SCS)

The self-control scale published by Tangney et al. in 2004 is divided into a full version and a short version (Tangney et al., 2004). We chose to use the full version, which includes 36 items and five dimensions. Those dimensions were self-discipline, non-impulsive action, healthy habits, work ethic, and reliability. Alpha values and test-retest reliability for the total self-control scale were both.89. Using this scale, studies found that impaired self-control ability was one of the characteristics of substance addicts (Michael, 2000). In the current study, the Cronbach α of the total scale was 0.764.

### Statistical Methods

Data analysis was conducted in SPSS version 22.0 and Mplus version 7.0. The significance level was set to p < 0.05. The BIS/BAS scales were evaluated for reliability using internal consistency, split-half reliability, and test-retest reliability. Internal consistency was evaluated using Cronbach’s alpha coefficient. The split-half reliability and test-retest reliability were evaluated using Spearman correlation coefficients. The construct validity was analyzed using confirmatory factor analysis. We used the chi-squared, comparative fit index (CFI), Tucker-Lewis index (TLI), the root mean square error of approximation (RMSEA), and standardized root mean square residual (SRMR) to evaluate the fit of each model. CFI and TLI greater than 0.90, and RMSEA and SRMR less than 0.08 indicate an acceptable model fit (Sun, 2005). All items obtained significant skewness and kurtosis values (*p* < 0.001) in the Kolmogorov-Smirnov normality test, which meant the data did not fit the normal distribution; therefore, so the robust maximum likelihood with standard error and mean adjustments (MLM) estimator was used in all confirmatory factor analyses (CFAs) (DiStefano, 2002). It is acceptable for the factor loading of the item to be greater than 0.3 (Wang, 2014). The convergent and discriminant validity and subscale inter-correlations were evaluated using the Spearman correlation coefficients, then the difference in correlation coefficients was analyzed using *cocor*, a free software package for the R programming language (Birk et al., 2015). The Mann-Whitney U test and independent sample ratio difference significance test (Zhang and Xu, 2015) were used to evaluate the differences between the two groups. The data did not conform to normal distribution; therefore, it was presented as rank mean when performed the Mann-Whitney *U* test.

## Results

### Basic Demographic Data

The average age of the people with a substance use disorder and community residents were 35.88 (SD: 8.19 years) and 38.06 (SD: 11.35 years), and 66.2% and 46.00% of them were male, respectively. The unemployment rate among the participants with a substance use disorder was much higher than among community participants. Most participants with a substance use disorder were educated to the primary and middle levels. A total of 907 participants with a substance use disorder reported using ice drug, and polydrug abuse was common. See Table 1 for specific information.

**TABLE 1:** Demographic data of people with a substance use disorder (n = 1117) and community residents (*n* = 822) in this study.

	Participants with a substance use disorder (n/%)	Community residents (n/%)
**Gender**		
Male	740 (66.2%)	378 (46.0%)
Female	377 (33.8%)	444 (54.0%)
**Age** (M ± SD)	35.88 ± 8.19	38.06 ± 11.35
**Employment**		
Unemployed	478 (42.8%)	49 (5.9%)
Employed	591 (52.9%)	773 (94.0%)
**Educational level**		
≤Primary	181 (16.2%)	27 (3.3%)
Middle	571 (51.1%)	158 (19.2%)
High	321 (28.7%)	154 (18.7%)
≤University	9 (0.8%)	483 (58.8%)
**Category ^a^**		
Ice drug	907 ^b^	
Magu	732^b^	
Heroin	280^b^	
Ketamin	191^b^	
mada	144^b^	

*^a^This category showed the top five drugs used by most people; ^b^The number of people mentioned had taken this drug.*

### Confirmatory Factor Analysis of the BIS/BAS Scales

We performed a confirmatory factor analysis on the four-factor model of Carver and White, as well as the one-factor model (Müller and Wytykowska, 2005) and two-factor model (Ross et al., 2002), five-factor model (Johnson et al., 2003; Heym et al., 2008). The five-factor model proposed by Johnson et al. used items 2 and 22 as the fear subscale and the remaining items in the BIS as the anxiety subscale. The five-factor model proposed by Heym et al. used items 2, 22, and 16 as the fear subscale and the remaining items in the BIS as the anxiety subscale. The remaining dimensions were consistent with the four-factor model proposed by Carver and White. The results showed that the five-factor model fitted to the data. The results are shown in Table 2. Factor loadings for CFA of factor models are shown in Table 3.

**TABLE 2:** Fit indices of existing models of the BIS/BAS scales in people with a substance use disorder (*n* = 1117).

	χ^2^/df	CFI	TLI	RESEA	SRMR
One factor	1340.565	0.705	0.671	0.079	0.075
Two factors	1041.120	0.780	0.753	0.068	0.073
Four factors	763.955	0.849	0.825	0.057	0.064
**Five factors(Johnson)^∗^**	462.173	0.923	0.907	0.042	0.041
Five factors(Heym)	747.573	0.852	0.824	0.057	0.063

* Items 2 and 5 correlated; Items 11 and 13 correlated; and Items 10 and 14 correlated; CFI = comparative fit index; TLI = Tucker-Lewis index; RMSEA = root-mean-square error of approximation; SRMR = standardized root mean square residual.

**TABLE 3:** Factor loadings for Confirmatory factor analysis of five factor models.

Variable	One factor	Two factors	Four factors	Five factors (Johnson)	Five factors (Heym)
Item 3	0.481	0.519	0.636	0.557	0.636
Item 9	0.576	0.614	0.74	0.659	0.741
Item 12	0.512	0.534	0.523	0.552	0.522
Item 21	0.509	0.533	0.596	0.611	0.596
Item 5	0.487	0.498	0.526	0.527	0.522
Item 10	0.387	0.392	0.49	0.438	0.495
Item 15	0.437	0.431	0.522	0.476	0.530
Item 20	0.560	0.573	0.649	0.669	0.643
Item 4	0.459	0.466	0.453	0.444	0.456
Item 7	0.590	0.593	0.614	0.607	0.614
Item 14	0.628	0.615	0.654	0.652	0.651
Item 18	0.456	0.433	0.482	0.486	0.482
Item 23	0.532	0.508	0.570	0.575	0.572
Item 8	0.431	0.504	0.516	0.518	0.514
Item 13	0.369	0.597	0.593	0.587	0.589
Item 16	0.415	0.620	0.610	0.603	0.612
Item 19	0.349	0.532	0.539	0.534	0.539
Item 24	0.409	0.585	0.580	0.584	1.176
Item 2	–0.088	0.090	0.101	0.441	0.077
Item 22	–0.167	0.054	0.055	0.547	0.084

### Inter-Factor Correlations and Reliability

The inter-scale correlation coefficient of the scale was between -0.216 and 0.519. There were significant positive correlations between the three subscales of BAS and also between two subscales of BIS. Three subscales of BAS were significantly negatively correlated with BIS-fear and positively correlated with BIS-anxiety. The internal consistency coefficient ranged from 0.387 to 0.824, both of which were within the acceptable range, and only the internal consistency coefficient of BIS-fear was relatively low. The test-retest reliability of the total scale was 0.532. The split-half reliability was 0.652. Specific information is illustrated in Table 4.

**TABLE 4:** Inter-factor correlations and internal consistency of BIS/BAS scales on five-factor model in participants with a substance use disorder (*n* = 1117).

	M ± SD	BAS-fun seeking	BAS-reward	BIS-fear	BIS-anxiety	Cronbach’s alpha
BAS-drive	8.705 ± 2.079	0.473**	0.519**	−0.216**	0.211**	0.711
BAS-fun seeking	8.897 ± 1.936		0.456**	−0.179**	0.339**	0.634
BAS-reward	9.751 ± 2.174			−0.113**	0.461**	0.690
BIS-fear	5.324 ± 1.096				0.110**	0.387
BIS-anxiety	10.526 ± 2.177					0.699
BIS	15.850 ± 2.56					0.624
BAS	27.352 ± 5.047					0.824
BIS/BAS	43.202 ± 6.272					0.808

** p < 0.01, * p < 0.05.

### Convergent and Discriminant Validity

The correlation coefficient between SR and three subscales of BAS was markedly larger than that between two subscales of BIS (*p* < 0.05). The correlation coefficient between SP and two subscales of BIS was markedly larger than that between three subscales of BAS (*p* < 0.05). In addition, we also found that the correlation coefficient between three subscales of BAS and SR was markedly larger than that between SP (*p* < 0.05). The correlation coefficient between two subscales of BIS and SP was markedly larger than that between SR (*p* < 0.05). The SUPPS-P correlated more substantially with three subscales of BAS than that with two subscales of BIS (*p* < 0.05, only the difference of correlation coefficient between BAS-reward and BIS-fear was not significant). The fun seeking dimension correlated moderately with the SCS. The results are shown in Table 5.

**TABLE 5:** Spearman correlations among BIS/BAS scales and other measures in people with a substance use disorders (*n* = 362).

	SP	SR	SCS	S-UPPS-P
M ± SD	35.52 ± 5.030	35.02 ± 4.930	111.36 ± 13.206	53.81 ± 5.028
BAS-drive	0.003	0.431**	0.122*	0.324**
BAS-fun seeking	0.211**	0.358**	0.472**	0.444**
BAS-reward	0.140**	0.394**	0.190**	0.190**
BIS-fear	0.386**	0.190**	0.109*	0.082
BIS-anxiety	0.337**	0.063	0.146**	–0.047
BIS	0.447**	0.162**	0.161**	0.018
BAS	0.142**	0.475**	0.316**	0.381**
BIS/BAS	0.284**	0.456**	0.329**	0.324**
SP		0.374**	0.334**	0.165**
SR			0.193**	0.342**
SCS				0.312**
S-UPPS-P				

** p < 0.01, * p < 0.05; SP = The punishment subscales of the punishment and reward sensitivity scales; SR = The reward subscales of the punishment and reward sensitivity scales; SCS = The self-control scale; S-UPPS-P = The short version of the UPPS-P impulsive behavior scale.

### Comparison Between Participants With a Substance Use Disorder and Community Residents

Based on the BIS and BAS scores, 27% of the people at each end were allocated into four groups: BIS high and BAS high score group; BIS high and BAS low score group; BIS low and BAS high score group; and BIS low and BAS low score group. In the comparison of the five dimensions, the score for people with a substance use disorder in the fun seeking dimension was significantly lower than that of the community residents. Second, the score for people with a substance use disorder in the reward responsiveness and BIS-fear dimensions were significantly higher than that of the community residents. There were no significant differences in the remaining dimensions. In the comparison of the four groups, there were significantly fewer people with high BAS activity and low BIS activity among the people with a substance use disorder than among the community residents. There were significantly more people with high BAS activity and high BIS activity among the people with a substance use disorder than among the community residents. There were no significant differences among the remaining groups. The results are shown in Table 6.

**TABLE 6:** Comparisons of participants with a substance use disorder and community residents of four subscales.

	People with a substance use disorder (n = 1117)	Community residents (n = 822)	|Z|
BAS-fun seeking	933.56	1019.52	3.378**
BAS-reward	1075.41	826.77	9.745**
BAS-drive	969.93	970.09	0.006
BIS-fear	1000.01	929.22	2.838**
BIS-anxiety	968.51	972.51	0.171
BIS high and BAS high score group	151	95	1.282
BIS high and BAS low score group	57	119	7.101**
BIS low and BAS high score group	55	30	1.356
BIS low and BAS low score group	121	44	4.281**

* p < 0.05; ** p < 0.01; Scores for five dimensions are presented as rank mean in rows 2–5; The number of people in four group showed in rows 6–9.

## Discussion

Since Carver et al. developed the BIS/BAS scales in 1994, its factor structure has ignited controversy. The four-factor structure proposed by Carver et al. was validated in most CFA studies (Moreira et al., 2015; Maack and Ebesutani, 2018). Meanwhile, many scholars have proposed some new structures, such as the five-factor structure mentioned above. In the present study, the five-factor model was the best fit for people with a substance use disorder; however, it failed to meet the psychometric standard. We correlated items 3 and 9; items 10 and 15; and items 4 and 5 to modified model. All of the modifications were based on the MI applied by Mplus and had sufficient theoretical meaning because the two pairs of correlated items have similar directionality and were loaded on the same factor in our study. We found the descriptions of item 3 and item 9, item 10 and item 15 were similar, meanwhile they were both loaded on drive and fun-seeking dimensions, respectively. For item 4 and item 5, both of them expressed individual’s willingness of doing something. In previous studies, these two items have also been correlated (Yu et al., 2011). People who were undergoing treatment in compulsory detoxification institutions usually had poor education and might confuse the meaning of two items. The factor loading of each item ranged from 0.438 to 0.669, which was within the acceptable range. It also indicated that the five-factor model was more suitable for people with a substance use disorder.

The factor structure of BIS has been controversial in previous research. Some researchers have confirmed that BIS consists of BIS-fear and BIS-anxiety dimensions (Caci et al., 2007), while other scholars support the one-factor structure of BIS (Muris et al., 2005). This question was controversial for the following reasons (Maack and Ebesutani, 2018). Firstly, a single factor is simpler and easier to explain than two factors; secondly, the appearance of BIS-fear factor was caused by a lack of modeling method effects or reverse scoring. Generally, our study provided empirical support for the substantive significance of BIS-fear factor and two-factor structure of BIS, which was consistent with johnson et al.’s study (Johnson et al., 2003).

The result of inter-factor correlation was consistent with previous studies (Heubeck et al., 1998; Caci et al., 2007). The internal consistency coefficient was only low for BIS-fear dimension. Correlation analysis found that the correlation coefficient between two items was relatively low (0.235). Dissabandara also reported low alpha levels for the BIS-Fear scale in heroin-dependent samples [α = 0.17, (Dissabandara et al., 2012)]. Low test-retest reliability might be associated with a series of negative symptoms experienced during substance withdrawal, such as restlessness, anxiety (Benowitz, 2008) and increased response time (Snyder et al., 1989). These symptoms might have some influence on the questionnaire filling.

SPSRQ, like the BIS/BAS scales, is a self-reporting scale that measures RST (Torrubia et al., 2001). The correlation between those two scales was consistent with the results of previous studies (Caseras et al., 2003), and indicated that the BIS/BAS scales had good convergent and discriminant validity. Previous studies always associated impulsivity with BAS activity. For example, Caseras et al. found that all three BAS subscales were associated with measures of impulsivity, among which the fun-seeking subscale was the most prominent. We had similar findings in the present study. The relationship between BAS and impulsivity has always been an issue worth studying. BAS was initially proposed as the causal basis of impulsivity (Smillie et al., 2006), while others argued that impulsive behavior did not stem intrinsically from BAS (Patock-Peckham et al., 2001). No causal conclusion can be drawn in the present study, and its further mechanism depends on future research. The relationship between self-control and the fun seeking dimension could be explained by the negative effects of the use of self-control (Hagger et al., 2010). Exercising self-control is a difficult thing. In particular, when people do not receive the corresponding reward, they are likely to pursue more enjoyable behavior. Another possible explanation for this results centers on the temporary decrease in the sensitivity of behavioral inhibition system caused by self-control (Inzlicht and Gutsell, 2007). BAS may play a leading role in this situation. In summary, the above results indicated that BIS was different from BAS. BIS was more sensitive to punishment and BAS was more closely related with reward sensitivity and impulsivity.

Surprisingly, we found participants with a substance use disorder were significantly insensitive compared with the community residents in the reward responsiveness dimension. As typically found, people with a substance use disorder have a heightened sensitivity to cues associated with BAS (Dissabandara et al., 2012). This could be explained by the interaction between BAS and BIS. Some participants with a substance use disorder might be less sensitive in the reward responsiveness dimension than the community residents; however, their BIS might be more insensitive. Therefore, BIS, which has the function of suppressing prepotent conflicting behaviors and resolving goal conflict (Corr, 2004), may be not work properly, making it difficult for people with a substance disorder to weigh the pros and cons and correctly resolve the conflict. The association between BIS and substance use was observed to be inconsistent in previous studies. Previous studies had failed to find significant differences in BIS dimensions between people with heroin or cocaine addiction and the healthy control group (Franken et al., 2006; Dissabandara et al., 2012), while Loxton et al. found that club-drug users had significantly lower BIS scale scores than non-drug users (Loxton et al., 2008). These different findings may be related to different types of drugs used.

Contrary to our expectations, we found that there were significantly fewer people with a substance use disorder than community residents in BIS low and BAS high sensitivity group. The first thing to note is that this does not mean that the community residents had higher levels of BAS and lower levels of BIS than people with a substance use disorder. According to the joint subsystems hypothesis (Corr, 2002), reactivity also changes because of the nature of the stimulus. Corr found that low anxiety/high impulsivity participants were least reactive to unpleasant slides. Therefore, it can be inferred that community residents have a less emotional response and decreased risk of taking impulsive behavior to conflicts than people with a substance use disorder. Considering the huge difference in their living environment, we thought that it would be sensible to analyze the sensitivity of BIS and BAS in combination with the effect of learning and experience. There was evidence that the activation levels of both systems was affected by learning (Smillie et al., 2007). BAS-reactive individuals should display superior learning when their good behavior is rewarded (compared to those with a less reactive BAS). Winning experience might affect the promotion of BAS, which takes charge of the experience of positive feelings and the hindrance of BIS, which is responsible for the experience of negative feelings (Kim and Lee, 2011). Thus, community residents with good experience would more likely to choose behaviors that conform to social expectations. In the high anxiety/high impulsivity group, Corr put proposed that participants showed attenuated fear potentiation. Analyzing the characteristics of BIS and BAS in people with a substance use disorder was more complex. This involved not only the euphoria of the drug (aversive conditioned stimuli), but also a sense of failure to control oneself (appetitive conditioned stimuli). That is to say, attenuated fear potentiation might be the result of mutual interplay of the BAS and BIS under two stimuli of different properties. Corr also found that participants high anxiety/high impulsivity were more reactive to negative stimuli than to positive stimuli, which revealed that people with a substance use disorder were more likely to make high-risk choices. Generally, the above results supported joint subsystems hypothesis. Influenced by the nature of stimulation and daily experience, there were differences in personality traits and behavioral tendencies between the two groups.

## Conclusion

The present study found that the BIS/BAS scales had good applicability in Chinese participants with a substance use disorder who were undergoing treatment in compulsory detoxification institutions. In addition, this study supported joint subsystems hypothesis and indicated that people with a substance use disorder were more likely to take impulsive and risky behavior than community residents. However, this study also had some shortcomings. First, this study failed to distinguish the differences in BIS and BAS levels caused by different drugs. Second, the two groups of subjects could not be completely matched demographically because of certain practical reasons, such as participants under compulsory rehabilitation usually having poor education and low income, and there were always more male than female participant with a substance use disorder. In addition, this study did not conduct long-term follow-up surveys of the subjects and could not provide dynamic data. In short, this study proved that BIS/BAS scales have satisfactory psychometric properties in people with a substance use disorder and enriched our knowledge of the personality characteristics of people with a substance use disorder and general population.

## Data Availability Statement

The datasets generated for this study are available on request to the corresponding author.

## Ethics Statement

The studies involving human participants were reviewed and approved by the Medical Ethics Committee of the Second Xiangya Hospital, Central South University (Changsha, China). The patients/participants provided their written informed consent to participate in this study.

## Author Contributions

QC, PY, HG, JZ, and TC conceived and designed the study. QC, PY, and ML organized and supervised collection and input of data. QC and PY drafted the manuscript, organized and supervised the data analysis. HG, ML, JZ, and TC provided critical comments on various drafts of the manuscript. All authors read and approved the final manuscript.

## Conflict of Interest

The authors declare that the research was conducted in the absence of any commercial or financial relationships that could be construed as a potential conflict of interest.

## Abbreviations

The BIS/BAS scales: the Behavioral Inhibition/Behavioral Activation Scales
BIS: Behavioral Inhibition system
BAS: Behavioral Activation system
SPSRQ: The punishment and reward sensitivity scales
SCS: The Self-control scale
SUPPS-P: The Short version of the UPPS-P Impulsive Behavior Scale.
